# Calf circumference predicts changes of bone mineral density in postmenopausal osteoporotic women receiving denosumab

**DOI:** 10.1007/s40520-025-02989-7

**Published:** 2025-05-05

**Authors:** Cecilia Oliveri, Anastasia Xourafa, Nunziata Morabito, Adele Di Giovanni, Elisa Lupo, Giorgio Basile, Agostino Gaudio, Antonino Catalano

**Affiliations:** 1 Unit and School of Geriatrics, Department of Clinical and Experimental Medicine, University Hospital of Messina, Via C. Valeria, 98125 Messina, Italy; 2 Unit of Talassemia, University Hospital of Catania, Catania, Italy; 3 Department of Clinical and Experimental Medicine, University Hospital of Catania, Catania, Italy

**Keywords:** Muscle mass, Muscle strength, Bone mineral density, Osteoporosis, Denosumab, Fracture risk

## Abstract

**Background:**

Aging is associated with deterioration of muscle and bone health, resulting in increased fragility fracture risk. It is not known whether muscle mass and strength could impact the osteoporosis pharmacological response.

**Aim:**

The aim of this study was to analyze the association between muscle mass and strength with the response to denosumab in osteoporosis.

**Methods:**

Postmenopausal women at high fracture risk receiving denosumab (60 mg subcutaneously administered every 6 months) were considered. The likelihood of sarcopenia was estimated by administering the SARC-F questionnaire, muscle mass and performance were assessed by measuring calf circumference (CC) and hand grip strength, respectively. Bone mineral density (BMD) was measured by dual energy X-ray absorptiometry.

**Results:**

130 women (age 70.2 ± 9.4 years) were recruited. Baseline BMD T-score values were − 2.6 ± 1.1 SD and − 2.3 ± 0.7 SD at lumbar spine and femoral neck, respectively; while CC and grip strength were 31.9 ± 2.9 cm and 22.7 ± 6.7 kg, respectively. The SARC-F score was associated with the 10-year probability of major osteoporotic fracture (r = 0.21, p < 0.05). The CC was positively associated with the T-score values of both lumbar spine (r = 0.262, p = 0.034) and femoral neck (r = 0.359, p = 0.004). Denosumab administration (treatment duration 43 months), lead to BMD improvement by + 9.6% at the lumbar spine and + 7.3% at the femoral neck (p^all^ < 0.05). After adjustment for comorbidities, fracture risk and treatment duration, the CC (β = 1.76, SE = 0.82, p = 0.03) and the baseline femoral BMD (β = − 94.19, SE = 26.09, p = 0.0009) were independently associated with femoral BMD gain over time.

**Conclusion:**

In postmenopausal osteoporotic women, the CC was positively and independently associated with denosumab treatment response.

## Introduction

Fragility fractures represent a major public health concern responsible for increasing social costs [1].

Osteoporosis is the most common systemic bone disease characterized by reduced bone mass and disruption of bone architecture, resulting in increased risk of fragility fractures, which represent the main clinical consequence of the disease. Due to the aging population, the number of incident fractures related to osteoporosis is estimated to rise significantly in the next years [2].

Muscle health is closely associated to bone strength; hence, low bone mass often coexists with reduced muscle mass, in part due to common risk factors and pathophysiology for bone and muscle disorders, as seen in osteosarcopenia that represent a relatively new geriatric giant [3].

Accordingly, low physical activity may negatively impact muscle and bone [4]; conversely, physical exercise may preserve musculoskeletal health [5, 6]. Moreover, mounting evidence suggests a crosstalk between muscle and bone tissues leading to mutual metabolic influence [7]. In fact, some muscle-derived molecules, such as the myokines irisin and the exercise-induced muscle factor β-aminoisobutyric acid, positively influence bone tissue by enhancing osteoblast activity and preventing osteocyte apoptosis; conversely, some signals derived from bone affects either positively the skeletal muscle, such as osteocalcin, which recovers muscle functions during exercise, or negatively, such as nuclear factor-kappa B receptor activating ligand (RANKL), which decreases muscle function and strength [8].

It has also been observed that patients diagnosed with sarcopenia are twice as likely to have osteoporosis as healthy subjects, and that for a one-unit increase in skeletal muscle mass there is a 36% reduction in the risk of osteoporosis [4].

Calf circumference (CC) represents an anthropometric measurement correlated with muscle mass [9]. Consistently, a decreased CC was observed in subjects diagnosed with sarcopenia [10–12].

CC, as well as body mass index (BMI), has been inversely associated with fragility hip fracture rate among community-dwelling older adults [13].

Handgrip strength is also associated with muscle mass, walking speed, balance and activities of daily living [14].

Larger CC and higher handgrip strength have been associated with better gait speed reserve that is a valuable marker of muscle-skeletal health [15]. Contrariwise, a smaller CC, as well as reduced grip strength, has been correlated with lower bone mineral density (BMD), supporting the close relationship between muscle and bone health [16]. However, no evidence is available on their predictive value as a marker of response to treatment with anti-osteoporotic drugs.

Denosumab (Dmab) is a monoclonal antibody able to inhibit the RANKL, preventing osteoclasts proliferation and activity, thus exerting a bone antiresorptive activity. It was proven to safely increase BMD and reduce fracture risk in different clinical settings [17–20]. Experimental data also suggest that Dmab could improve muscle strength and have a positive impact on muscle performance in patients with low BMD [21, 22].

To our knowledge, the impact of muscle mass and strength in predicting the efficacy of Dmab has not been studied in real life.

The main aim of this study was to explore the impact of CC, as a simple anthropometric measure of muscle mass, and handgrip strength, as a reliable indicator of muscle performance, with the change of BMD occurring under treatment with Dmab for severe osteoporosis.

## Methods

This retrospective study included postmenopausal women at high risk of fracture who presented to an outpatient tertiary care clinic for metabolic bone disease between the years 2020 and 2023. Patients with a history of cancer, renal or liver failure, heart failure, thyroid or parathyroid dysfunction or abnormal serum calcium levels were not included. Participants were patients who had been prescribed Dmab for the secondary prevention of fragility fractures, in accordance with the national drug reimbursement legislation in Italy (https://www.aifa.gov.it/documents/20142/1728074/nota-79.pdf). Measurement of BMD in the six months prior to the start of Dmab and at the end of the study was mandatory for patient eligibility, as was a minimum duration of treatment with Dmab of eighteen months without discontinuation.

At the beginning of Dmab treatment, the fracture risk assessment tool, FRAX, available at http://www.shef.ac.uk/FRAX was used to calculate the 10-year probability of major fractures (which included fractures of the hip, spine, humerus or wrist) and hip fractures, taking into account several clinical risk factors such as age, sex, weight, height, personal fracture history, parental hip fracture history, smoking habit, glucocorticoid use, rheumatoid arthritis, secondary osteoporosis, alcohol intake). The FRAX score was computed without including BMD [23].

Comorbidities were taken into account and expressed through the Charlson Comorbidity Index (CCI) score [24]. The SARC-F questionnaire (Strength, Assistance in walking, Rise from a chair, Climb stairs, and Falls) was conducted to investigate the probability of sarcopenia (for a score ≥ 4): the result was obtained by assigning each of the five items investigated a score from 0 to 2 [25]. Calf circumference (CC) was measured at baseline in a sitting position, with the knee flexed to 90°, using a tape measure. The measurement was taken at the point with the largest diameter, recording the maximum value between the two limbs, expressed in cm [26]. A Jamar dynamometer was used to assess the hand grip strength, expressed in kg [27]. BMD was measured by dual-energy X-ray absorptiometry (DXA), gold-standard, on the lumbar spine (L1–L4) in anteroposterior projection and on the femoral neck, using a Hologic Discovery densitometer calibrated daily with a coefficient of variation (CV) of 0.5%. Morphometric vertebral fractures were assessed by radiography of the spine according to Genant's criteria, as previously described [28, 29]. Blood samples for measurements of vitamin D [25(OH)D] status, surrogate biomarkers of bone remodeling, including C-terminal collagen type 1 telopeptide (CTX) and alkaline phosphatase (ALP), phosphorus, calcium and creatinine were taken in the morning after an overnight fast, before the first Dmab administration. Dmab at a dose of 60 mg was subcutaneously injected every 6 months to all the participants who received also 25,000 IU cholecalciferol every 14 days. Only patients with inadequate dietary calcium intake received daily calcium carbonate supplementation of 500–1000 mg.

The MedCalc software (version 10.2.0.0, Mariakerke, Belgium) was used to perform statistical analyses. The normal distribution of the data was determined by the Kolmogorov–Smirnov test. Data were reported as mean ± SD. The Pearson’s correlation coefficient was calculated to measure the correlation between variables, and multiple regression was performed to analyze the association between a dependent variable and one or more explanatory variables. Particularly, we performed a model by considering the time change in femoral neck BMD as a dependent variable, while age, BMI, CCI, CC, handgrip strength, femoral neck BMD at baseline, 25(OH)D and duration of Dmab treatment as independent variables. A p value < 0.05 indicated statistical significance for all the tests used.

## Results

The main clinical characteristics of the participants (n = 130) are listed in Table 1. The mean age of the recruited women was 70.2 ± 9.4 years, and the mean Dmab treatment duration was 43.2 ± 24 months. As highlighted in Table 1, participants showed a high fracture risk with a 10-year probability of major osteoporotic fractures and hip fracture of 29.3% and 14.6%, respectively. CCI score (4.4 ± 1.7) was indicative of a population with comorbidities. SARC-F greater than 4 was encountered in 42 (32.3%) patients. These participants when compared with those with SARC-F values lower than 4, showed significantly higher age (72.7 ± 10.5 vs 68.02 ± 9.31 year), higher CCI (5.48 ± 2.06 vs 3.79 ± 1.29), higher FRAX derived 10-year probability of hip fractures (18.96 ± 14.3 vs 12.97 ± 13.2%) and lower handgrip strength score (19.09 ± 6.37 vs 24.77 ± 6.4 kg).

**Table 1:** Baseline clinical characteristics of participants

	Participants (n = 130)
*Main clinical characteristics*	
Age (year)	70.2 ± 9.4
BMI (kg/m^2^)	24.7 ± 5
SARC-F score	3.4 ± 2.4
Calf circumference (cm)	31.9 ± 2.9
Handgrip strength (kg)	22.7 ± 6.7
Charlson comorbidity index	4.4 ± 1.7
10-year probability of major fractures (%)	29.3 ± 13.2
10-year probability of hip fractures (%)	14.6 ± 12.9
Dmab treatment duration (months)	43.2 ± 24
*Laboratory data*	
25(OH)D (ng/mL)	42.6 ± 16.1
Serum creatinine (mg/dL)	0.82 ± 0.24
Serum calcium (mg/dL)	9.32 ± 0.23
Serum phosphorus (mg/dL)	3.16 ± 0.21
Alkaline phosphatase (U/L)	71.2 ± 43.1
CTX (ug/L)	0.32 ± 0.28
*DXA measurements*	
L1-L4 BMD (g/cm^2^)	0.75 ± 0.1
Femoral neck BMD (g/cm^2^)	0.59 ± 0.1
L1-L4 T-score (SD)	−2.6 ± 1.1
Femoral neck T-score (SD)	−2.3 ± 0.7

Data are expressed as mean ± SD

*BMD* bone mineral density, *25(OH)D* 25-hydroxyvitamin D, *CTX* C-terminal collagen type 1 telopeptide

The CCI was positively correlated with the FRAX score calculated for major osteoporotic fractures (r = 0.391, p < 0.001) and for hip fractures (r = 0.51, p < 0.001). The CCI score was negatively associated with vitamin D status, as measured by 25(OH)D levels (r = − 0.19; p = 0.02) and with the handgrip strength (r = −0.36; p < 0.001); therefore, those with more comorbidities were at high fracture risk and showed lower vitamin D levels and less muscle strength. The CCI score was also positively associated with chronological age (r = 0.618, p < 0.001) and BMI (r = 0.242, p = 0.008). The SARC-F score was positively associated with age (r = 0.243, p = 0.022), CCI score (r = 0.482, p < 0.001), FRAX score (both for major fractures: r = 0.21; and for femoral fracture: r = 0.047, p^all^ < 0.05) and change in lumbar BMD (r = 0.25, p = 0.05); it has been negatively associated with the handgrip (r = −0.473, p < 0.001). The CC was positively associated with T-score values at lumbar spine (r = 0.262, p = 0.034) and femoral neck (r = 0.359, p = 0.004) (Figs. 1, 2).

The CC was positively correlated with BMI (r = 0.44, p < 0.001) and negatively correlated with the FRAX score (r = −0.275, p = 0.028 and r = − 0.246, p = 0.04; for major osteoporotic and hip fractures, respectively). Handgrip strength was also negatively associated with age (r = − 0.368, p =  < 0.001) and with the 10-year risk of femur fracture (r = − 0.219, p = 0.015); a positive association with the BMD value at the femoral neck was also observed at the end of the study (r = 0.212, p = 0.05).

No participants reported fractures during the observation period. At the end of the study, BMD increased both at the lumbar spine (T-score: − 2.24 ± 1.13 vs − 2.76 ± 0.97 SD), and at the femoral neck (T-score: − 2.08 ± 0.78 vs − 2.32 ± 0.77 SD).

The absolute change in BMD was 9.6% and 7.3% for lumbar spine and femur, respectively. BMD changes at lumbar spine and femoral neck were inversely associated with the respective baseline BMD values (r = − 0.37, p = 0.001 and r = − 0.41, p < 0.001). Femoral neck BMD modification was also inversely associated with CCI (r = − 0.27, p = 0.012). At a multiple regression analysis, the femoral neck BMD change was considered as a dependent variable, while age, BMI, CCI, CC, handgrip strength, femoral neck BMD at baseline, 25(OH)D and duration of Dmab treatment as independent variables: in this model, only CC (β = 1.76, SE = 0.82, p = 0.03) and the baseline femoral BMD (β = − 94.19, SE = 26.09, p = 0.0009) were independently associated with changes in femoral BMD over time.

## Discussion

This study investigated for the first time the influence of muscle mass and performance on the BMD modification occurring under Dmab treatment in a setting of postmenopausal women suffering from osteoporosis.

The close relation between bone and muscle mass is a matter of fact both in physiologic and pathological condition [30]. The prevalence of osteosarcopenia is commonly higher in older people who show common risk factors for developing osteoporosis and sarcopenia, including physical inactivity and poor nutrition, but also suffer the higher comorbidity burden.

The reduced muscle function is also associated with disuse osteoporosis, as observed in the hemiparetic limb after stroke or in genetically determined conditions such as muscular dystrophy, [31, 32].

The impaired muscle mass and/or function could therefore affect bone metabolism, not only because of reduced mechanical stress, but also due to the modification of molecular patterns related to the release of cytokines and myokines [33, 34].

Recently, bi-directional biochemical communication between muscle and bone has been highlighted and there is growing interest in muscle-derived molecules that can drive bone metabolism and maintain bone health [33]. The story of irisin is an example of the recent advancement in this field [35, 36]. Irisin is an exercise-induced hormone that promotes osteogenesis and mitigates bone loss in osteoporosis and sarcopenia and is independently associated with vertebral fragility fractures [37, 38].

However, the impact of muscle mass/strength on the results of anti-osteoporosis treatment has not yet been studied. In the present study, participants were older women with high risk of fragility fracture in whom the higher fracture risk was associated with the lower CC and handgrip strength, in agreement with the literature data. Lower handgrip strength was also observed in women with higher comorbid burden, the latter being also related to poorer vitamin D status. So, vitamin D confirmed its role as marker of general health status [39], but also as possible pathophysiologic link between multimorbidity and fragility fracture by conditioning low muscle strength [40, 41]. The largest CC was similarly measured in participants with the higher BMD, this association being consistently maintained when considering both the lumbar spine and the femoral neck.

Dmab significantly improved BMD at the lumbar spine and femoral neck, in association with the administration of vitamin D and by guarantying adequate calcium intake in all the participants. The BMD gain was achieved independently of patient’s age and BMI and was significantly and independently associated with the baseline BMD, meaning that participants with lower BMD were those who reported the greatest BMD improvement at the end of the study. However, the main clinical predictor of femoral BMD improvement caused by denosumab was CC. In this study, CC captured also the influence of comorbidities as suggested by the multivariate analysis we performed. Beyond the speculative observation a woman with higher muscle mass may be prone to higher physical activity also promoting bone health, a possible action of Dmab on muscle has been proposed too. Rupp et al. reported that Dmab improved muscle strength at the limbs in osteoporotic women [21] and consistently reduced the propensity to fall [42]. Recently, it has been proposed that the effect on muscle of Dmab may be independent of the action on bone, being dependent on RANKL blockade, and may be, at least in part, responsible for the observed reduction of fracture risk [43].

Overall, this study confirms the relevance of both femoral neck BMD and calf circumference in predicting the response to Dmab: since lower femoral BMD may be observed in women with lower limb muscle mass, it may be speculated that in these patients the Dmab efficacy may be also carried out by a beneficial action on muscle tissue, finally leading to fracture risk reduction.

While we confirmed the association between muscle strength and fracture risk in postmenopausal women, we previously observed [27, 44], an association between Dmab response and baseline handgrip strength was not observed in this study. Although this result may be taken as unexpected, it could be related to the restricted precision in capturing the muscle performance of the lower limbs by the handgrip measurement, compared to other performance tests such as, for example, the sit-to-stand test, we not performed [21]. Dmab may also have an effect on musculature, as suggested by the reduced propensity to fall of treated patients, which may contribute to the reduction of fracture risk by the use of Dmab, as indicated by the post-hoc analysis of five clinical studies involving Dmab performed by Chotiyarnwong et al. [42].

We should acknowledge our study has some limitations, mainly represented by the retrospective design, the small sample size, the participation of only women which does not allow to extend the results obtained to men, and the lack of other muscle functional tests, in addition to the measurement of the handgrip, able to explore the performance of the lower limbs. At the same time, the homogeneity of the sample, represented only by postmenopausal women, all receiving the same treatment, including vitamin D, and the centralized instrumental and clinical measurements, may be considered as research’s strengths.

## Conclusions

This study highlights the sustained improvement of BMD caused by Dmab in a real-world scenario, focusing on postmenopausal women at high fracture risk. BMD change, at lumbar spine and femoral neck, was associated with muscle mass, and the larger BMD gain at femoral neck was obtained in women showing the wider calf circumference. These findings may stimulate clinicians to pay attention to muscle in the personalized approach to osteoporosis management and may also encourage further prospective studies looking at muscle to finally promote fracture risk reduction.

## Data Availability

No datasets were generated or analysed during the current study.

## References

[1] Hernlund E, Svedbom A, Ivergård M et al (2013) Osteoporosis in the European Union: medical management, epidemiology and economic burden. A report prepared in collaboration with the International Osteoporosis Foundation (IOF) and the European Federation of Pharmaceutical Industry Associations (EFPIA). Arch Osteoporos 8(1):136. 10.1007/s11657-013-0136-1 10.1007/s11657-013-0136-1 PMC3880487 24113837

[2] Willers C, Norton N, Harvey NC et al (2022) Osteoporosis in Europe: a compendium of country-specific reports. Arch Osteoporos 17:23. 10.1007/s11657-021-00969-8 35079919 10.1007/s11657-021-00969-8 PMC8789736

[3] Veronese N, Ragusa FS, Sabico S et al (2024) Osteosarcopenia increases the risk of mortality: a systematic review and meta-analysis of prospective observational studies. Aging Clin Exp Res 36:132. 10.1007/s40520-024-02785-9 38888670 10.1007/s40520-024-02785-9 PMC11189340

[4] He H, Liu Y, Tian Q et al (2016) Relationship of sarcopenia and body composition with osteoporosis. Osteoporos Int 27:473–482. 10.1007/s00198-015-3241-8 26243357 10.1007/s00198-015-3241-8

[5] Izquierdo M, de Souto BP, Arai H et al (2024) Global consensus on optimal exercise recommendations for enhancing healthy longevity in older adults (ICFSR). J Nutr Health Aging. 10.1016/j.jnha.2024.100401 39743381 10.1016/j.jnha.2024.100401 PMC11812118

[6] Gaudio A, Rapisarda R, Xourafa A et al (2021) Effects of competitive physical activity on serum irisin levels and bone turnover markers. J Endocrinol Investig 44:2235–2241. 10.1007/s40618-021-01529-0 33675533 10.1007/s40618-021-01529-0 PMC8421288

[7] Herrmann M, Engelke K, Ebert R et al (2020) Interactions between muscle and bone-where physics meets biology. Biomolecules 10:432. 10.3390/biom10030432 32164381 10.3390/biom10030432 PMC7175139

[8] Colaianni G, Storlino G, Sanesi L et al (2020) Myokines and osteokines in the pathogenesis of muscle and bone diseases. Curr Osteoporos Rep 18:401–407. 10.1007/s11914-020-00600-8 32514668 10.1007/s11914-020-00600-8

[9] Rolland Y, Lauwers-Cances V, Cournot M et al (2003) Sarcopenia, calf circumference, and physical function of elderly women: a cross-sectional study. J Am Geriatr Soc 51:1120–1124. 10.1046/j.1532-5415.2003.51362.x 12890076 10.1046/j.1532-5415.2003.51362.x

[10] Kawakami R, Murakami H, Sanada K et al (2015) Calf circumference as a surrogate marker of muscle mass for diagnosing sarcopenia in Japanese men and women. Geriatr Gerontol Int 15:969–976. 10.1111/ggi.12377 25243821 10.1111/ggi.12377

[11] Champaiboon J, Petchlorlian A, Manasvanich BA et al (2023) Calf circumference as a screening tool for low skeletal muscle mass: Cut-off values in independent Thai older adults. BMC Geriatr 23:826. 10.1186/s12877-023-04543-4 38066438 10.1186/s12877-023-04543-4 PMC10709895

[12] Kim HJ, Kim JY, Kim SH (2024) Performance of calf circumference in identifying sarcopenia in older patients with chronic low back pain: a retrospective cross-sectional study. BMC Geriatr 24:674. 10.1186/s12877-024-05263-z 39127641 10.1186/s12877-024-05263-z PMC11316404

[13] Azevedo DC, Hoff LS, Kowalski SC et al (2024) Risk factors for osteoporotic hip fracture among community-dwelling older adults: a real-world evidence study. Adv Rheumatol 64:8. 10.1186/s42358-024-00350-6 38233892 10.1186/s42358-024-00350-6 PMC13488741

[14] Inoue H, Hayashi Y, Watanabe H et al (2023) Handgrip strength is correlated with activities of daily living, balance, and body composition in patients with thoracolumbar compression fracture. Medicine (Baltimore) 102:e33141. 10.1097/MD.0000000000033141 36862919 10.1097/MD.0000000000033141 PMC9981377

[15] Lindholm B, Basna R, Ekström H et al (2024) Gait Speed Reserve in the general population-based “Good Aging in Skåne” cohort study-distribution and associated factors. Geroscience. 10.1007/s11357-024-01318-6 39192005 10.1007/s11357-024-01318-6 PMC11872813

[16] Arazi H, Eghbali E, Saeedi T et al (2016) The relationship of physical activity and anthropometric and physiological characteristics to bone mineral density in postmenopausal women. J Clin Densitom 19:382–388. 10.1016/j.jocd.2016.01.005 26922458 10.1016/j.jocd.2016.01.005

[17] Cummings SR, San Martin J, McClung MR et al (2009) FREEDOM Trial. Denosumab for prevention of fractures in postmenopausal women with osteoporosis. N Engl J Med 361(8):756–65. 10.1056/NEJMoa0809493. 10.1056/NEJMoa0809493 19671655

[18] Bone HG, Wagman RB, Brandi ML et al (2017) 10 years of denosumab treatment in postmenopausal women with osteoporosis: results from the phase 3 randomised FREEDOM trial and open-label extension. Lancet Diabetes Endocrinol 5:513–523. 10.1016/S2213-8587(17)30138-9 28546097 10.1016/S2213-8587(17)30138-9

[19] Catalano A, Oliveri C, Natale G et al (2024) Renal function is associated with changes in bone mineral density in postmenopausal osteoporotic women treated with denosumab: data from a retrospective cohort study. J Clin Med 13:6239. 10.3390/jcm13206239 39458189 10.3390/jcm13206239 PMC11514604

[20] Diab DL, Watts NB (2024) The use of denosumab in osteoporosis—an update on efficacy and drug safety. Expert Opin Drug Saf 23:1069–1077. 10.1080/14740338.2024.2386365 39262109 10.1080/14740338.2024.2386365

[21] Rupp T, von Vopelius E, Strahl A et al (2022) Beneficial effects of denosumab on muscle performance in patients with low BMD: a retrospective, propensity score-matched study. Osteoporos Int 33:2177–2184. 10.1007/s00198-022-06470-3 35751664 10.1007/s00198-022-06470-3 PMC9546982

[22] Bonnet N, Bourgoin L, Biver E et al (2019) RANKL inhibition improves muscle strength and insulin sensitivity and restores bone mass. J Clin Investig 129:3214–3223. 10.1172/JCI125915 31120440 10.1172/JCI125915 PMC6668701

[23] Catalano A, Morabito N, Basile G et al (2013) Fracture risk assessment in postmenopausal women referred to an Italian center for osteoporosis: a single day experience in Messina. Clin Cases Miner Bone Metab 3:191–194 (**PMID: 24554930**) PMC3917582 24554930

[24] Novella A, Elli C, Tettamanti M et al (2021) Comparison between drug therapy-based comorbidity indices and the Charlson Comorbidity Index for the detection of severe multimorbidity in older subjects. Aging Clin Exp Res 33:1929–1935. 10.1007/s40520-020-01706-w 32930989 10.1007/s40520-020-01706-w

[25] Beaudart C, McCloskey E, Bruyère O et al (2016) Sarcopenia in daily practice: assessment and management. BMC Geriatr 16:170. 10.1186/s12877-016-0349-4 27716195 10.1186/s12877-016-0349-4 PMC5052976

[26] Landi F, Onder G, Russo A et al (2014) Calf circumference, frailty and physical performance among older adults living in the community. Clin Nutr 33:539–544. 10.1016/j.clnu.2013.07.013 23948128 10.1016/j.clnu.2013.07.013

[27] Catalano A, Gaudio A, Bellone F et al (2022) Trabecular bone score and phalangeal quantitative ultrasound are associated with muscle strength and fracture risk in hemodialysis patients. Front Endocrinol (Lausanne) 13:940040. 10.3389/fendo.2022.940040 36157439 10.3389/fendo.2022.940040 PMC9489856

[28] Genant HK, van Kuijk C, Jergas M (1995) Vertebral fracture in osteoporosis. Radiology Research and Education Foundation, San Francisco

[29] Atteritano M, Mirarchi L, Venanzi-Rullo E et al (2018) Vitamin D status and the relationship with bone fragility fractures in HIV-infected patients: a case control study. Int J Mol Sci 19:119. 10.3390/ijms19010119 29301284 10.3390/ijms19010119 PMC5796068

[30] Kaji H (2014) Interaction between muscle and bone. J Bone Metab 21:29–40. 10.11005/jbm.2014.21.1.29 24707465 10.11005/jbm.2014.21.1.29 PMC3970293

[31] Marzolini S, McIlroy W, Tang A et al (2014) Predictors of low bone mineral density of the stroke-affected hip among ambulatory individuals with chronic stroke. Osteoporos Int 25:2631–2638. 10.1007/s00198-014-2793-3 25001986 10.1007/s00198-014-2793-3

[32] Catalano A, Vita GL, Russo M et al (2016) Effects of teriparatide on bone mineral density and quality of life in Duchenne muscular dystrophy related osteoporosis: a case report. Osteoporos Int 27:3655–3659. 10.1007/s00198-016-3761-x 27589974 10.1007/s00198-016-3761-x

[33] Dong Y, Yuan H, Ma G et al (2024) Bone-muscle crosstalk under physiological and pathological conditions. Cell Mol Life Sci 81:310. 10.1007/s00018-024-05331-y 39066929 10.1007/s00018-024-05331-y PMC11335237

[34] Gaudio A, Pennisi P, Bratengeier C et al (2010) Increased sclerostin serum levels associated with bone formation and resorption markers in patients with immobilization-induced bone loss. J Clin Endocrinol Metab 95:2248–2253. 10.1210/jc.2010-0067 20305005 10.1210/jc.2010-0067

[35] Gaudio A, Rapisarda R, Zanoli L et al (2021) Effects of competitive physical activity on serum irisin levels and bone turnover markers. J Endocrinol Investig 44:2235–2241. 10.1007/s40618-021-01529-0 33675533 10.1007/s40618-021-01529-0 PMC8421288

[36] Battafarano G, Rossi M, Marampon F et al (2020) Bone control of muscle function. Int J Mol Sci 21:1178. 10.3390/ijms21041178 32053970 10.3390/ijms21041178 PMC7072735

[37] Colaianni G, Mongelli T, Cuscito C et al (2017) Irisin prevents and restores bone loss and muscle atrophy in hind-limb suspended mice. Sci Rep 7:2811. 10.1038/s41598-017-02557-8 28588307 10.1038/s41598-017-02557-8 PMC5460172

[38] Palermo A, Strollo R, Maddaloni E et al (2015) Irisin is associated with osteoporotic fractures independently of bone mineral density, body composition or daily physical activity. Clin Endocrinol (Oxf) 82:615–619. 10.1111/cen.12672 25400208 10.1111/cen.12672

[39] Martino G, Catalano A, Bellone F et al (2018) Quality of life in post-menopausal women: which role for vitamin D? Mediterr J Clin Psychol 6:1–14. 10.6092/2282-1619/2018.6.1875

[40] Catalano A, Bellone F, Santoro D et al (2021) Vitamin D boosts alendronate tail effect on bone mineral density in postmenopausal women with osteoporosis. Nutrients 13:1878. 10.3390/nu13061878 34072655 10.3390/nu13061878 PMC8226654

[41] Conzade R, Grill E, Bischoff-Ferrari HA et al (2019) Vitamin D in relation to incident sarcopenia and changes in muscle parameters among older adults: the KORA-Age Study. Calcif Tissue Int 105:173–182. 10.1007/s00223-019-00558-5 31069442 10.1007/s00223-019-00558-5

[42] Chotiyarnwong P, McCloskey E, Eastell R et al (2020) A pooled analysis of fall incidence from placebo-controlled trials of denosumab. J Bone Miner Res 35:1014–1021. 10.1002/jbmr.3972 31999376 10.1002/jbmr.3972 PMC9328365

[43] Gostage J, Kostenuik P, Goljanek-Whysall K et al (2024) Extra-osseous roles of the RANK-RANKL-OPG axis with a focus on skeletal muscle. Curr Osteoporos Rep 22:632–650. 10.1007/s11914-024-00890-2 39325366 10.1007/s11914-024-00890-2 PMC11499344

[44] Catalano A, Sardella A, Bellone F et al (2020) Executive functions predict fracture risk in postmenopausal women assessed for osteoporosis. Aging Clin Exp Res 32:2251–2257. 10.1007/s40520-019-01426-w. (**Epub 2019 Nov 26. PMID: 31773488**) 31773488 10.1007/s40520-019-01426-w

